# Antioxidants Characterization of the Fruit, Juice, and Pomace of Sweet Rowanberry (*Sorbus aucuparia* L.) Cultivated in Estonia

**DOI:** 10.3390/antiox10111779

**Published:** 2021-11-06

**Authors:** Viive Sarv, Petras Rimantas Venskutonis, Reelika Rätsep, Alar Aluvee, Rita Kazernavičiūtė, Rajeev Bhat

**Affiliations:** 1 Polli Horticultural Research Centre, Institute of Agricultural and Environmental Sciences, Estonian University of Life Sciences, Uus 2, Polli, Mulgi Parish, 69108 Viljandi, Estonia; rimas.venskutonis@ktu.lt (P.R.V.); reelika.ratsep@emu.ee (R.R.); Alar.Aluvee@emu.ee (A.A.); 2 ERA Chair for Food (By-) Products Valorisation Technologies (VALORTECH), Estonian University of Life Sciences, Fr. R. Kreutzwaldi 1, 51006 Tartu, Estonia; rajeev.bhat@emu.ee; 3 Department of Food Science and Technology, Kaunas University of Technology, Radvilėnų˛ pl. 19, LT-50254 Kaunas, Lithuania; rita.kazernaviciute@ktu.lt

**Keywords:** antioxidants, polyphenolic compounds, rowanberry pomace, hybrid cultivars

## Abstract

This study aimed to identify promising candidates of rowanberry cultivars for a wider cultivation and utilization. Antioxidant properties and phenolic content were evaluated for fruit, juice, and pomace samples of 16 different sweet rowanberry cultivars (cvs) and wild rowanberry (*S. aucuparia* L.), while the antioxidant potential was assessed using three different methods, based on the capacity to scavenge ABTS^●+^ and DPPH^●^ and measure the oxygen radical absorbance capacity (ORAC). In general, the radical scavenging capacity was higher for hybrid cultivars, e.g., for cvs Likernaja, Burka, Granatnaja, and Rubinovaja in all assays. The highest value in the ABTS^●+^ assay was determined for the fruit sample Likernaja, and in DPPH^●^ assay in the pomace sample of cv. Likernaja, at 527.55 and 1068.28 µM TE/g dw, respectively. The highest ORAC value was found in the fruit sample of Burka (456.53 µM TE/g dw). Among the Nevezhino rowans, the highest radical scavenging values of all fractions were determined in cv. Solnechnaja. Regarding the total phenolic content (TPC), higher values were obtained in the whole fruits than in separated fractions, juice, and pomace. The tested hybrids had higher TPC values, either in fruit and pomace or in juice extracts, than those in the other analyzed *S. aucuparia* L. cultivars. While the fruit and juice samples showed higher anthocyanin (ACY) values, the pomace samples had higher hydroxycinnamic acid (HCA) contents on average. The results revealed that the different fractions of selected rowanberry cultivars can be a promising source of antioxidants and polyphenols for further potential applications. It is envisaged that the results of this study will serve in valorizing sweet rowanberry cultivars as value-added functional ingredients for food and non-food applications.

## 1. Introduction

According to the recent report by Grand View Research, Inc., the global market of polyphenols is predicted to reach USD 2.08 billion by 2025 [1]. These compounds have demonstrated antioxidant, anti-inflammatory, anti-diabetic, anti-diarrheal, anti-tumor, as well as diuretic and vasodilatory effects. Many fruits and particularly berries are superior sources of polyphenols with a high antioxidant capacity [2,3]. Therefore, fruit-origin raw materials have been growingly utilized to extract bioactive compounds for various applications. In some cases, the processing of fruits generates a substantial number of by-products [4]. For example, fruit pomace, which is a solid residue of juice pressing, consists mainly of skin, seeds, and pulp, and it accounts for approximately 10–35% of the mass of the initial fresh fruit [4]. Moreover, the pomace holds a considerable number of polyphenolic compounds, approximately 28–35% in the skin, 60–70% in seeds, and 10% in pulp, making it a potential source of natural antioxidants [5,6]. Although, many research articles have been published on the valorization of by-products from agro-industry, including fruit pomace [4], juice pressing residues of some fruit remain under-investigated.

Rowan is a fairly common fruit crop in different countries of the world. The orange or reddish fruits of *Sorbus aucuparia* L. are small (diameter 6–9 mm) and they have been traditionally used as diuretic, laxative, anti-inflammatory, and vasoprotective agents, against rheumatism and kidney diseases as well as for the treatment of various gastrointestinal and respiratory tract-related disorders [7].

**Figure 1:**
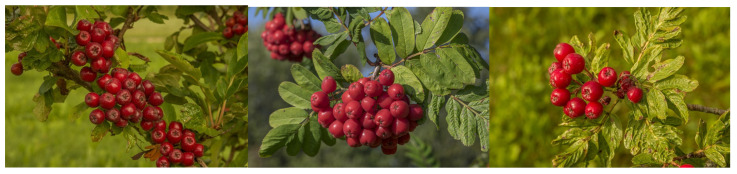
Rowanberry cultivars ‘Likernaja’, ‘Alaja Krupnaja’ and ‘Granatnaja’.

Although the rowanberries have been used for juice, jams, or jellies [8,9], their application for foods is limited due to their bitter and astringent taste. To overcome this hindrance, the first sweet rowanberry clones were selected from the Sudety Mountains (Czech Republic) already in the 19th century. At the beginning of the 20th century, Russian scientist and plant breeder Michurin started a breeding program of sweet rowanberries for northern conditions and developed the most interesting group of *S. aucuparia* hybrids with *Pyrus, Malus, Aronia,* or *Crataegus* species [10]. The taste of the cultivated hybrid fruits such as Likernaja, Alaja Krupnaja, and Granatnaja (Figure 1), is less astringent, and the fruits are usually larger and darker in color than those of wild rowanberries [9,11]. The varieties Kubovaya, Zheltaya, and Krasnaya were selected from the sweet-fruited form of *S. aucuparia* originated from the village Nevezhino in Russia, while the varieties Rossica and Rosina were bred of the Moravian mountain ash from the Sudety Mountains. Regarding the quality characteristics of rowanberries, Bussinka, Vefed, and Solnechnaja were rich in vitamin C content, while the latter two were also not astringent [12]. Moreover, previous investigations have reported the antioxidant capacity [3] and bacteriostatic effect [13] of both wild and cultivated rowanberry extracts.

Considering the diverse genetic background of sweet rowanberry cultivars, there is no comprehensive information available about the antioxidant properties and phenolic content of these fruit, juice, and pomace. Therefore, the antioxidant capacity, phenolic content, and phytochemicals of 16 sweet rowanberry cultivars: cvs Burka, Alaja Krupnaja, Granatnaja, Kubovaja, Rosina, Rubinovaja, Angri, Bussinka, Likernaja, Moravica, Oranzhevaja, Krasnaja, Sahharnaja, Solnechnaja, Rossica, and Vefed, were determined using in vitro assays. It is expected that the results of this study will serve in valorizing sweet rowanberry cultivars as value-added functional ingredients for food and non-food applications.

## 2. Materials and Methods

The chemicals in procedures were analytical grade and purchased from Sigma-Aldrich (Steinheim, Germany).

### 2.1. Preparation of Sweet Rowanberry Samples

Ripe fruit of 16 sweet rowanberry cultivars (Table 1) and wild rowanberry were harvested in autumn 2019 from Polli Experimental Station, Estonia. All fruits were immediately frozen and stored at −20 °C. The low-speed juicer Smeg SJF01CREU (Smeg S.p.A, Guastalla, Italy) was used to extract the juice from defrosted fruit. The remaining pomace accounted for approximately 15–20% of the weight of the fresh rowanberries. The rowanberries, juice, and pomace were frozen at −40 ± 2 °C and freeze-dried in an Advantage Plus Benchtop Freeze Dryer (SP Industries, Warminster, PA, USA) for 72 h at 30 µbar. The pomace samples were ground in a Retsch Mixer Mill M 400 (Haan, Germany) for 1.5 min at 30 Hz using ZrO_2_ balls. The lyophilized rowanberries, pomace, and juice were stored in hermetically closed bottles at −25 °C.

### 2.2. Determination of Antioxidant Capacity

Antioxidant capacity was measured using four methods, based on the rowanberry phytochemicals to reduce Folin–Ciocalteu’s reagent (generally called as total phenolic content, TPC), their ability to scavenge 2,2′-azino-bis(3-ethylbenzo-thiazoline-6-sulfonic acid radical cation (ABTS^●+^) and stable diphenyl-picrylhydrazyl radical (DPPH^●^), as well as oxygen radical absorbance capacity (ORAC). The experts have previously used at least 3 of these methods, including TPC and ORAC, for a comprehensive evaluation of the antioxidant potential of natural products [18]. All spectrophotometric measurements of juice and fruit samples and ORAC of pomace were performed on a FLUOstar Omega Microplate Reader (BMG Labtech, Offenburg, Germany); TPC and ABTS^●+^/DPPH^●^ scavenging values of pomace samples prepared by QUENCHER method were determined on a Spectronic Genesys 8 spectrophotometer (Thermo Spectronic, Rochester, NY, USA). TPC was expressed as gallic acid equivalents in grams of dry sample weight (mg GAE/g), radical scavenging in Trolox equivalents (mg TE/g) unless indicated differently. All described measurements in this section were replicated four times.

Antioxidant capacity was measured using four methods, based on the rowanberry phytochemicals to reduce Folin–Ciocalteu’s reagent (generally called as total phenolic content, TPC), their ability to scavenge 2,2′-azino-bis(3-ethylbenzo-thiazoline-6-sulfonic acid radical cation (ABTS^●+^) and stable diphenyl-picrylhydrazyl radical (DPPH^●^), as well as oxygen radical absorbance capacity (ORAC). The experts have previously used at least 3 of these methods, including TPC and ORAC, for a comprehensive evaluation of the antioxidant potential of natural products [18]. All spectrophotometric measurements of juice and fruit samples and ORAC of pomace were performed on a FLUOstar Omega Microplate Reader (BMG Labtech, Offenburg, Germany); TPC and ABTS^●+^/DPPH^●^ scavenging values of pomace samples prepared by QUENCHER method were determined on a Spectronic Genesys 8 spectrophotometer (Thermo Spectronic, Rochester, NY, USA). TPC was expressed as gallic acid equivalents in grams of dry sample weight (mg GAE/g), radical scavenging in Trolox equivalents (mg TE/g) unless indicated differently. All described measurements in this section were replicated four times.

#### 2.2.1. Sample Preparation

Freeze-dried juice samples were dissolved in methanol (MeOH, 1 *w*/*v*) by treating 15 min in the ultrasound bath. Then, the solutions were centrifuged at 4500 rpm for 5 min and transparent centrifugate was used directly for measurements. It was decided to apply the QUENCHER procedure for measuring the antioxidant capacity of freeze-dried fruit and pomace. This method enables the determination of antioxidant capacity both of bound and free radical scavengers [18]. However, grinding of the freeze-dried fruit was rather complicated, most likely due to the presence of viscous pectic substances; therefore, 1 g of fruit was homogenized with 10 mL of MeOH at 9500 rpm during 1 min in IKA T 25 digital ULTRA-TURRAX Disperser (IKA^®^-Werke GmbH & Co. KG, Staufen, Germany). The homogenate was centrifuged at 12,000 rpm for 5 min and the supernatant was collected, diluted to the required concentration, and used for analysis.

The preparation of pomace samples in the QUENCHER procedure was carried out as described by Serpen et al. [18] with some modifications. The stock mixture was produced by mixing freeze-dried pomace with microcrystalline cellulose at a ratio of 1:1 (*w*/*w*). Afterward, a series of “solid dilutions” of stock mixture with microcrystalline cellulose was performed to obtain the concentrations in the range of 1–40 µg/mg. Based on these results, 10 mg of freeze-dried pomace were used in all assays.

#### 2.2.2. Total Phenolic Content (TPC)

The TPC was measured with Folin–Ciocalteu’s reagent as originally described by Singleton et al. [19]. Briefly, 30 μL of juice or fruits sample was mixed with 150 μL of 10-fold diluted with distilled water Folin−Ciocalteu reagent and 120 μL of 7.5% Na_2_CO_3_ in microplate wells. After mixing, the microplate was placed in the FLUOstar Omega Reader, shaken for 30 s, incubated for 30 min at room temperature, and the absorbance was read at 765 nm wavelength. All measurements were replicated four times. A blank sample, which was prepared daily, contained the same amount of distilled water. A series of gallic acid solutions in the concentration range of 0.025–0.35 mg/mL was used for the calibration curve (regression equation: *y* = 9.8307*x* + 0.1215, *R*^2^ = 0.9987). In the case of pomace (QUENCHER approach) 10 mg of sample or cellulose (blank) were mixed with 150 µL of distilled H_2_0, 750 µL of Folin-Ciocalteu’s reagent, and 600 µL of Na_2_CO_3_ solution, vortexed for 15 s, shaken at 250 rpm for 2 h in the dark, centrifuged (4500 rpm, 5 min) and the absorbance of optically clear supernatant was measured at 760 nm. Gallic acid solutions (150 µL) at various concentrations (0–80 µg/mL) were used for calibration.

#### 2.2.3. DPPH^●^ Scavenging Capacity

DPPH^●^ scavenging capacity (RSC) of extracts was determined by a slightly modified spectrophotometric method of Brand-Williams et al. [20]. The aliquots of dissolved juice and fruits extracts (7.5 μL, 0.1%) were mixed in a FLUOstar Omega 96 well microplate reader with 300 μL of DPPH^●^. The decrease of absorbance was measured at 515 nm by comparing it with a blank. The final RSC values were calculated by using a regression equation *y* = 275.34*x* + 5.4266 (*R*^2^ = 0.99), which was obtained by using different concentration solutions of Trolox for building the calibration curve. The antioxidant capacity of each sample is expressed as mg of Trolox equivalent (TE) per g of dry weight sample.

Pomace or cellulose (blank) were transferred to a centrifugation tube, mixed with 500 μL of MeOH and 1000 μL of working DPPH^•^ solution, vortexed for 15 s, shaken at 250 rpm for 2 h in the dark, centrifuged (4500 rpm, 5 min), and the absorbance of optically clear supernatant was measured at 515 nm. Trolox solutions (25 μL) at various concentrations (0–1500 μmol/L MeOH) were used for calibration. For each well, an aliquot of 7.5 μL (0.1%) sample was mixed with 300 μL of DPPH^●^. The decrease of absorbance was measured at 515 nm by comparing it with a blank sample.

#### 2.2.4. ABTS^•+^ Scavenging Capacity

ABTS^•+^ decolorization assay was performed according to Re et al. [21], which is based on the reaction of ABTS^•+^ with antioxidants resulting in color change. The aliquots of dissolved juice and fruit extracts (3 μL, 0.1%) were mixed with 300 μL ABTS^•+^ solution in the microplate wells of FLUOstar Omega reader and the absorbance was measured at 734 nm against phosphate-buffered saline (PBS) solution, which was used as a blank. The final RSC values were calculated by using a regression equation *y* = 99.766*x* + 2.4483 (*R*^2^ = 0.99).

Pomace or cellulose (blank) were mixed with 25 μL of MeOH and 1500 μL of working ABTS^•+^ solution, vortexed for 15 s, shaken at 250 rpm for 2 h in the dark, centrifuged (4500 rpm, 5 min), and the absorbance of optically clear supernatant was measured at 734 nm. Trolox solutions (25 μL) at various concentrations (0–1500 μmol/L MeOH) were used for calibration.

#### 2.2.5. Oxygen Radical Absorbance Capacity (ORAC)

ORAC method was performed as described by Prior et al. [22] and Davalos et al. [23] by using fluorescein as a fluorescent probe. The stock solution of fluorescein was prepared according to Prior et al. [22]. The reaction was carried out in 75 mM phosphate buffer (pH 7.4), while the addition of antioxidant substances produced a more stable fluorescent signal which could reflect the antioxidant capacity.

For the subsequent assays, 25 μL (0.01%) of juice and fruit extract samples and 150 μL of PBS for fluorescein solution (95.68 nmol/L) were used. Solutions were placed in the 96 transparent flat-bottom microplate wells, the mixture was pre-incubated for 15 min at 37 °C, followed by rapid addition of AAPH solution as a peroxyl radical generator (25 μL; 240 mM) using a multichannel pipette. The microplate was immediately placed in the FLUOstar Omega reader, automatically shaken before each reading and the fluorescence was recorded every cycle (1 min × 1.1), a total of 120 cycles. The 485 nm excitation and 520 nm emission filters were used. At least three independent measurements were performed for each sample. Raw data were exported from the Mars software to Excel 2003 (Microsoft, Roselle, IL, USA) for further calculations. Antioxidant curves (fluorescence versus time) were normalized and from the normalized curves, the area under the fluorescence decay curve (AUC) was calculated as:

AUC = (1 + f_5_/f_0_ + f_6_/f_0_ + f_7_/f_0_ + ^…^+f_i_/f_0_);

where f_0_ = initial fluorescence reading at cycle 0, f_i_ = fluorescence reading at cycle i.

The final ORAC_FL_ values were calculated by using a regression equation (*y* = 0.1105*x* + 5.0662, *R*^2^ = 0.98) between Trolox concentration and AUC. The phosphate buffer saline (PBS) solutions of Trolox with known concentrations ranging from 5 to 250 µM/L were used for calibration. The antioxidant capacity in all assays is expressed as µM of Trolox equivalent (TE) per gram of dry weight sample.

Pomace or cellulose (blank) were mixed with 150 μL of PBS solution (75 mmol/L) and 900 μL of fluorescein solution (14 μmol/L PBS), vortexed for 15 s, shaken at 250 rpm for 60 min in the dark, and centrifuged (4500 rpm, 5 min). Optically clear supernatant (175 μL) was transferred to the 96-well black opaque microplates, pre-incubated for 15 min at 37 °C, followed by rapid addition of 25 μL of AAPH solution (240 mmol/L) as a peroxyl radical generator using a multichannel pipette. The fluorescence was recorded every cycle (1 min × 1.1), total of 90–140 cycles. Further experimental and data handling were the same as reported for extract analysis. Trolox solutions (150 μL) at various concentrations (0–500 μmol/L PBS) were used for calibration.

### 2.3. Identification and Quantification of Polyphenols by LC-MS Method

An ultra-high-performance liquid chromatography (UHPLC) was used for the analysis of individual phenolic compounds. Approximately 1 g of fresh fruit, juice, or pomace was mixed in 10 mL of 50% ethanol acidified with 1% of HCl. Before analysis, the samples were homogenized for 3 min using the IKA Ultra-Turrax^®^ Tube Drive (IKA^®^-Werke GmbH & Co. KG, Staufen, Germany) operating at 6000 rpm, followed by sonication at room temperature in an ultrasonic bath Branson 1800 (Emerson, St. Louis, MO, USA) for 15 min, and shaken in a multi-rotator Multi RS-60 (Biosan Sia, Riga, Latvia) for 30 min. Then, the samples were centrifuged at 13,000 rpm for 10 min (Eppendorf MiniSpin, rotor F-45–13.11) and 1 µL of extracts were pipetted into the vials for quantitative and qualitative chromatographic analysis, which was performed on UHPLC-DAD-LCMS 8040 (Shimadzu Nexera X2, Kyoto, Japan) using the reverse phase ACE Excel 3 C18-PFP column, 100 mm × 2.1 mm (ACE^®^ Advanced Chromatography Technologies Ltd., Aberdeen, Scotland), and pre-column SecurityGuard ULTRA, C18 (Phenomenex, Torrance, CA, USA) operating at 40 °C for the separation of individual polyphenols. The UHPLC system was equipped with a binary solvent delivery pump LC-30AD, an autosampler Sil-30AC, column oven CTO-20AC and diode array detector SPD-M20A. The flow rate of the mobile phase was 0.25 mL/min, and the injected sample size was 1 μL. Acidified (1% formic acid) mobile phases consisted of Milli-Q water (A) and methanol (B). Separation was carried out for 40 min under the following conditions: gradient 0–27 min, 10–80% B; 27–29 min, 80–95% B; 29–35 min, isocratic 95% B, and re-equilibration of the system with 10% B 8 min before the next injection. All samples were kept at 4 °C during the analysis.

The calibration ranges of standards were adjusted considering the estimated concentrations of polyphenolic compounds in the samples. Individual phenolic compounds were identified by comparing their retention times, UV spectra, and parent and daughter ion masses (m/z) with those of the reference compounds. MS data acquisitions were performed on LCMS 8040 with the ESI source operating in both positive and negative modes. The interface voltage was set to 4.5 kV (both ESI+ and ESI−). Nitrogen was used as the nebulizing gas (3 L/min) and drying gas (15 L/min). The heat block temperature was 350 °C and the desolvation line (DL) temperature was 250 °C. All the samples were analyzed in triplicate, and the results were expressed as milligrams per gram of dry weight.

### 2.4. Statistics

The mean values and standard deviations (SD) of ABTS^●+^/DPPH^●^ radical scavenging capacity (RSC) results and total phenolic contents (TPC) were calculated using MS Excel and one-way analysis of the variance (ANOVA) at *p* value < 0.05. Correlation coefficients (*R*^2^) between two RSCy assays and the polyphenolic groups were also calculated, using the statistical software from MS Excel.

## 3. Results and Discussions

### 3.1. Total Phenolic Content

**Figure 2:**
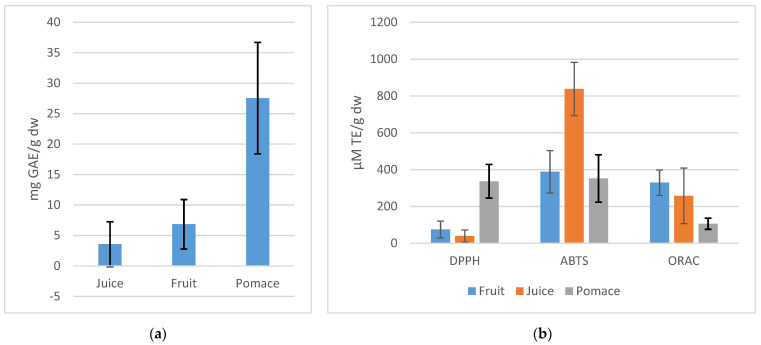
Mean values of TPC (**a**) and antioxidant capacity (**b**) of fruit, juice, and pomace of all cultivars in the current study.

The results obtained for TPC are depicted in Figure 2a. Accordingly, the pomace fraction has the highest mean value of TPC: compared to the mean value of fruit, it is four-fold, while the mean value of fruit, in turn, is two times higher than the TPC of juice. The standard deviation (SD) bars demonstrate the variety of TPC among the 16 cvs. An especially wide range of TPC is among the pomace part of cvs. These findings prove that the pomace part obtained from specific cvs can provide us a valuable source of polyphenols for food and pharmaceutical purposes [4,24].

As demonstrated in Table 2, the TPC values of 16 sweet rowanberry cvs ranged between 2.53 and 15.05 mg GAE/g dw, 0.53 and 14.8 mg GAE/g dw, and 15.97 and 44.68 mg GAE/g dw for whole fruit, juice, and pomace fractions, respectively. The highest levels were found for all fractions of cvs Likernaja, Burka, Rubinovaja, and Granatnaja. The cvs Likernaja and Burka are the hybrids between rowanberry and chokeberry, S. *aucuparia* × *Aronia melanocarpa,* and *Sorbus aria* × *Aronia arbutifolia*, respectively; while Rubinovaja is × *Sorbopyrus* (S. *aucuparia* × *Pyrus*) and Granatnaja is × *Sorbocrataegus* (S. *aucuparia* × *Crataegus*). The pomace fractions of the hybrids demonstrated the TPC values of 44.68 mg GAE/g dw for cvs Burka and 41 mg GAE/g dw for Likernaja and Rubinovaja. The TPC in the fruit of cv. Likernaja and cv. Burka was 15.05 and 14.78 mg GAE/g dw, respectively, while the contents in the juice of the same hybrids were 14.8 and 9.68 mg GAE/g dw, respectively. These results agree with the TPC values reported by Kampuse et al. [16] who found the highest TPC values for cv. Likernaja (484.9 mg/100 g fw) among the other 8 rowanberry cultivars. Hukkanen [14] tested many rowan cvs and found the highest TPC values for cvs Rubinovaja and Burka, 1014 and 820 mg/100 g of fw of fruit, respectively. In the research performed by Hukkanen et al., cv. Burka had the highest anthocyanin content among the sweet rowanberries. In the current research, the pomace fraction of cv. Moravica and wild rowanberry had very high TPCs, 29.32 and 31.7 mg GAE/g, respectively, while the highest TPCs among Nevezhino rowans were determined in the pomace of cv. Solnechnaja and Krasnaja, at 28.3 and 27.75 mg GAE/g dw, respectively. It may be observed that a significant fraction of polyphenols remains in the pomace, being the valuable part of rowanberries.

### 3.2. Antioxidant Capacity

The mean values of three antioxidant assays of rowanberry fruit, juice, and pomace (Figure 2b) demonstrate the different reaction mechanisms influencing these assays. Apak et al. [25] reported that although ABTS^•+^ reaction mechanism is still unclear, depending on individual antioxidants as well as reaction conditions, it is more a mixed-mode assay reagent, reacting by both ET (electron-) and HAT (hydrogen atom transfer) mechanisms. The DPPH^●^ is believed to act more like an H- atom acceptor, although the ET mechanism cannot be excluded, depending strongly on phenol-ionizing solvents and at alkaline pH where DPPH^●^ is a stable radical [25,26]. The ORAC assay is based on the HAT reaction mechanism [27].

The ABTS^●+^/DPPH^●^ scavenging and ORAC values are presented in Table 2. The DPPH^●^ scavenging activity ranged from 15.1 to 177.5 µM TE/g dw, 6.03 to 125.6 µM TE/g dw, and 172.1 to 527.6 µM TE/g dw, for fruit, juice, and pomace, respectively. Using ABTS^●+^ assay the antioxidant capacity values were between 666 and 1068 µM TE/g dw, 123.2 and 641.4 µM TE/g dw, and 179.9 and 584.2 µM TE/g dw for fruit, juice, and pomace, respectively. The results of ORAC assay ranged from 239.1 to 456.5 µM TE/g dw, 19.7 to 443.7 µM TE/g dw, and 43.87 to 150.8 µM TE/g dw, for fruit, juice, and pomace, respectively. All fractions of cvs Likernaja, Burka, Rubinovaja, and Granatnaja had the antiradical capacity values above the average. Comparing the pomace fractions, the cv. Likernaja presented the highest DPPH^●^ value of 527.55 µM TE/g dw, the cv. Burka had the highest ABTS^●+^ value of 576.77 µM TE/g dw, and the cv. Rubinovaja demonstrated the highest ORAC value of 150.75 µM TE/g dw. From previous studies, Jurikova et al. [16] and Kampuse et al. [28] found the highest antioxidant activity of cv. Likernaja which is among the other hybrids. Compared to the other cvs, all fractions of cv. Solnechnaja had very high ORAC values, as well as the DPPH^●^ and ABTS^●+^ values were above the average of 17 pomace samples. While the average ORAC and ABTS^●+^ values raise in the direction: pomace < juice < fruit, the rise of DPPH^●^ values is juice < fruit < pomace, and the average fruit and juice values of ABTS^●+^ are 10-fold compared to DPPH^●^ values. This phenomenon can be explained by the different reaction mechanisms in ABTS^●+^, DPPH^●^, and ORAC assays.

### 3.3. Identification and Quantification of Individual Phenolic Compounds in Different Fractions of Sweet Rowanberry Cultivars

**Figure 3:**
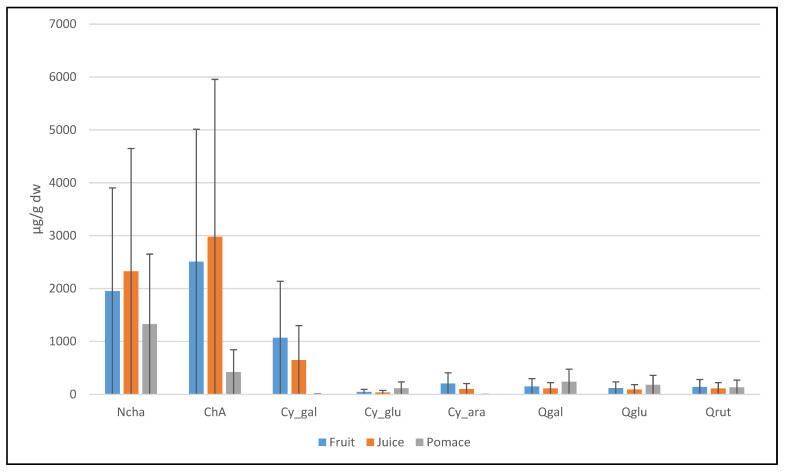
The mean contents of major polyphenolic compounds for all cultivars in current study.

The extracts recovered with acidified ethanol from fruit, juice, and pomace fractions were analyzed by UHPLC-DAD-MS/MS. The results (Figure 3 and Table 3) revealed that sweet rowanberry cvs are rich in caffeoylquinic acids, especially chlorogenic and neochlorogenic acids, ranging between 1.07 and 4.59 mg/g dw and between 0.75 and 6.13 mg/g dw, respectively. In our experiment, the highest contents of neochlorogenic acid were found in the fruit and juice samples of cvs Likernaja, Burka, Granatnaja, and Rubinovaja. The highest chlorogenic acid contents were determined in the fruit and juice samples of cvs Sahharnaja, Bussinka, Angri, and wild rowanberry. The neochlorogenic acids followed by chlorogenic acids were the most dominant phenolic acids in pomace samples (Figure 3). These findings were similar to the previous study of Bobinaitė et al. [29]. In the current study, the highest contents of neochlorogenic acid were tested in cvs Likernaja and Solnechnaja, but relatively high contents were determined also in cvs Burka, Bussinka and Granatnaja. Comparative data were reported by Jurikova et al., who found the highest content of chlorogenic acid in cvs Likernaja (100.9 mg/100 g fw) and Granatnaja (90.62 mg/100 g fw) [16]. While testing the chlorogenic acid content of the pomace samples, the highest values were found for wild rowanberry and cvs Bussinka and Sahharnaja, at 4.79 mg/g dw, 3.64 mg/g dw, and 3.62 mg/g dw, respectively. Mikulic-Petkovsek et al. [30] also reported cv. Bussinka to be rich in neochlorogenic acid.

Anthocyanins were the second most abundant group of polyphenols in sweet rowanberry cultivars. For instance, the fruits of cv. Burka had an even higher total ACY content (7.27 mg/g dw) than the content of total hydroxycinnamic acids (HCA), 5.10 mg/g dw. The other rowanberry hybrids, such as cvs Likernaja, Granatnaja, and Rubinovaja, also had relatively high total content of ACY, 6.33 mg/g dw, 3.20 mg/g dw, and 2.28 mg/g dw, respectively. The major part of ACY in the fruit and juice samples of hybrids was cyanidin-3-galactoside (up to 91% for Rubinovaja), followed by cyanidin-3-arabinoside (up to 21–22% for Likernaja and Burka). Cyanidin glucosides are a common group of anthocyanins in the rowanberries [30]. Kylli et al. [13] and Hukkanen et al. [14] also reported high contents of cyanidin-3-galactoside and cyanidin-3-arabinoside (together > 90% of the total ACYs) in the rowanberry hybrids. The fruit and juice of the other rowanberry cvs had ACY contents of less than 1 mg/g dw. Interestingly, in the case of rowanberry pomace, cyanidin-3-glucoside was the major part (up to 97%) of ACYs. Zymone et al. [15] and Mikulic-Petkovsec et al. [30] found cyanidin-3-galactoside to be the predominant anthocyanin in rowanberry pomace powder fruits. In our study, the highest total content of ACYs was found in pomace of cvs Burka and Likernaja, followed by cvs Rubinovaja and Granatnaja. The latter two are hybrid cultivars, originating from sweet rowanberries with intense dark colors.

The average content of ACYs was found up to 10-fold in the fruit and juice samples compared to that in pomace samples. At the same time, the average content of flavanols in the pomace samples was up to 4.8 times higher than that in the juice and fruit samples. In addition, the average contents of flavanols were lower in the fruit and juice samples than in the pomace samples.

**Figure 4:**
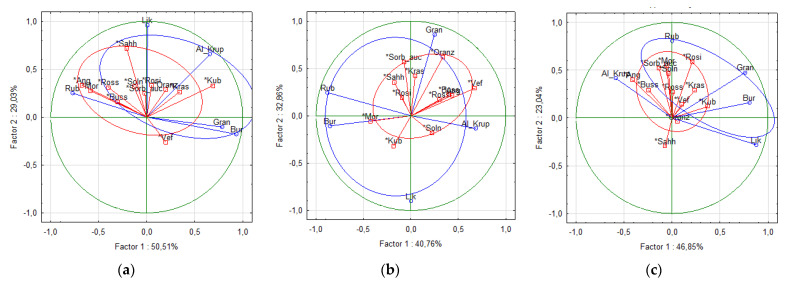
PCA score plots of different Sorbus fruit (**a**), juice (**b**), and pomace (**c**) samples.

A principal component analysis (PCA) of eight major phenolic compounds (Ncha, ChA, Cygal, Cyglu, Cyara, Qgal, Qglu, and Qrut) was conducted for the rowanberry fruit, juice, and pomace samples (Figure 4). All three (a, b, c) plots differentiated the cvs into two color-based groups, e.g., dark red hybrid cvs group (blue) and orange group of all other sweet rowanberry cvs (red). The first (a) plot, which illustrates the differentiation of fruit samples, had the highest score of two factors 79.54%, while the plot score of two factors for juice and pomace samples were 73.62% and 69.89%, respectively. The dark red hybrid samples have remarkably higher ACY content than the orange cvs, therefore, five hybrid samples located far from the 0-point of principal components, while most of orange-colored samples located nearby the 0-point of principal components due to more similar phytochemical compositions of these fruit.

Selecting the cvs with the best yield (years 2019 and 2021) and antioxidant capacity, four potential cvs among sixteen emerged. Therefore, hybrid cvs Likernaja and Burka, as well as Nevezhino rowans Sahharnaja and Solnechnaja, but also the wild rowanberry will be used in the further studies.

### 3.4. Correlation Analysis

The correlation analysis demonstrated the significant correlations between the ORAC, ABTS^●+^, and DPPH^●^ scavenging values and the main phenolic groups in the *Sorbus* fruit, juice, and pomace fractions. As presented in Table 4, relatively strong positive correlations were found between all antioxidant assays using the pomace, fruit, and juice extracts and their TPC (0.49 < *R*^2^ < 0.95) and ACY contents (0.48 < i^2^ < 0.89). The correlations between ORAC, ABTS^●+^, and DPPH^●^ scavenging values and FLAVO contents of three extracts was moderate (0.47 < *R*^2^ > 0.66), except the correlation between DPPH^●^ and FLAVO of fruit, which was weak (*R*^2^ = 0.28).

There was no correlation found between radical scavenging values and the contents of FLAVA in fruit, juice, and pomace extracts.

In the case of pomace extracts, the weak correlations were found between the radical scavenging values determined by ORAC, ABTS^●+^, and DPPH^●^ methods and the contents of HCA; however, there was a moderate correlation between the ORAC and ABTS^●+^ scavenging values and HCA content in the fruit, as well as between ABTS^●+^ and DPPH^●^ scavenging values and HCA content in juice extracts. The differences in correlations with polyphenolic groups and various radical scavenging methods, while using the same extracts, can be explained by the different reaction mechanisms in ORAC, ABTS^●+^, and DPPH^●^ assays, as described earlier (see Section 3.2).

The correlations between antioxidant assays and phenolic groups are different while using the whole fruit, pressed juice, or pomace for the analysis. In the current study working with 16 *Sorbus* cultivars and wild rowanberry, the major part (on average 85%) of the weight of fresh rowanberries comprised juice; therefore, it is expected that the fruit and juice could have comparable composition. The correlation analysis demonstrated comparable correlations between the antioxidant assays and polyphenolic groups of fruit and juice. The antioxidant activity of pomace samples, which consist mainly of peel and seeds, is influenced by TPC and ACY contents and moderately by FLAVO content in the samples, while in the case of the fruit and juice samples, HCA contents have an additional effect on radical scavenging values. Compared to the fruit and juice extracts, pomace extracts hold higher concentrations of protocatechuic acid and isorhamnetin, but also epicatechin, catechin, and procyanidins B1, B2, and C1, making the pomace fraction a considerable source of natural antioxidants.

## 4. Conclusions and Further Perspectives

The high yield and good antioxidant potential of starting materials were essential while selecting the potential cvs for total valorization. Therefore, the goal of the current study was the antioxidants characterization of the fruit, juice, and pomace of 16 best yielding sweet rowanberry (*Sorbus aucuparia* L.) cultivars and wild rowanberry grown in Estonia. Although 9 of 16 selected cvs and wild rowanberry were previously analyzed for polyphenolic content and antioxidant activity by different authors, it was relevant to compare the antioxidant characteristics of these best yielding cvs grown in different climatic conditions. Moreover, according to our knowledge the cultivar-based pomace characterizations have never been conducted.

In our study, twenty different phenolic compounds were detected in the acidified ethanolic extracts of cultivated and wild sweet rowanberry cultivars by UHPLC-MS. The contents of individual phenolic compounds in every investigated *S. aucuparia* L. cultivar, as well as the composition of rowanberry fruit, juice, and pomace samples, differed significantly from each other. In addition, the different constituents in the tested samples influenced the anti-radical scavenging activity in different fruit fractions of cultivars. Although the fruit and juice samples contain more ACYs than the pomace samples, the antioxidant characteristics of both are influenced by this group of polyphenols. On the other hand, the pomace samples, where the hydroxycinnamic acids dominated, were not affected by these components, and vice versa, the fruit and juice samples with lower HCA contents were more influenced by these polyphenolic acids. The fruit and juice samples of the sweet rowanberry hybrids Likernaja and Burka, crossbreeds with *Aronia melanocarpa* (Michx.) and *Aronia arbutifolia* L., respectively, had the highest contents of ACYs and HCAs. The pomace samples of the mentioned hybrids also had higher contents of ACYs when compared to the other investigated cultivars. As a significant part of phytochemicals remain in the rowanberry pomace fraction, it can be a potential source of functional ingredients for the biorefining process to increase the utilization of sweet rowanberry cultivars.

## Figures and Tables

**Table 1:** Description of selected sweet rowanberry *S. aucuparia* cultivars.

Cultivar	Origin	Breeding Background	Ref.
Burka	Russia, 1918	(*S. aria* × *Aronia arbutifolia* = *Sorbaronia alpina*) × *S. aucuparia*	[14,15]
Likernaja	Russia	S. *aucuparia* × *Aronia melanocarpa*	[15,16]
Granatnaja	Russia, 1925	*S. aucuparia* × *Crataegus sanguinea* = *Sorbocrataegus miczurinii*	[14,15]
Rubinovaja	Russia, 1927	*S. aucuparia* × *Pyrus communis* L.	[12,14,15]
Alaja Krupnaja	Russia, 1926	*S. aucuparia* × *Pyrus* sp. × *S. aucuparia* var. *moravica*	[14,15]
Moravica	Moravia, Check Republic, 19th cent.	The oldest cultivated *S. aucuparia*	[9,12]
Krasnaja	Nevezhino, Russia	Form of Nevezhino rowan (*S. aucuparia)*	[12,17]
Kubovaja	Nevezhino, Russia, 19th cent.	Form of Nevezhino rowan (*S. aucuparia)*	[12,14]
Oranzevaja	Nevezhino, Russia	Clone of Zheltaja, form of Nevezhino rowan (*S. aucuparia)*	[17]
Sahharnaja	Nevezhino, Russia	Form of Nevezhino rowan (*S. aucuparia)*	[12,17]
Vefed	Nevezhino, Russia	Cultivated form based on Nevezhino rowan (*S. aucuparia)*	[12]
Rossica	Germany, 1896	Clone of Moravica (*S. aucuparia)*	[12]
Solnechnaja	Nevezhino, Russia	Seedling of Kubovaja (*S. aucuparia)*	[12]
Angri	Nevezhino, Russia	Cultivated form based on Nevezhino rowan (*S. aucuparia)*	[12]
Bussinka	Nevezhino, Russia	Seedling of Kubovaja (*S. aucuparia)*	[12,15]
Rosina	Germany, 1946	Clone of Moravica (*S. aucuparia)*	[12,14]

**Table 2:** Total phenolic content, SET- and HAT-type antioxidant activity of fruit, juice, and pomace of 16 rowanberry genotypes and wild rowanberry.

		TPC			DPPH^●^			ABTS^●+^			ORAC	
	F	J	P	F	J	P	F	J	P	F	J	P
Bur	14.78 ± 1 ^a^	9.68 ± 1 ^b^	44.68 ± 2 ^a^	127.8 ± 9 ^b^	107.1 ± 4 ^b^	522.3 ± 36 ^a^	1010 ± 4 ^b^	641.4 ± 3 ^a^	576.8 ± 32 ^a^	456.5 ± 33 ^a^	435.7 ± 14 ^ab^	125.3 ± 8 ^abc^
Lik	15.05 ± 0 ^a^	14.8 ± 0 ^a^	41.31 ± 3 ^b^	84.38 ± 6 ^h^	125.61 ± 6 ^a^	527.6 ± 33 ^a^	1068 ± 8 ^a^	615.1 ± 9 ^a^	508.9 ± 27 ^b^	416.5 ± 29 ^ab^	381.9 ± 23 ^de^	128.5 ± 4 ^abc^
Gran	11.15 ± 1 ^b^	5.79 ± 0 ^c^	38.93 ± 3 ^c^	177.5 ± 3 ^a^	63.89 ± 4 ^c^	402.7 ± 22 ^c^	855.7 ± 1 ^d^	500.1 ± 4 ^b^	511.9 ± 35 ^b^	399.4 ± 31 ^cd^	396.8 ± 37 ^cd^	133.1 ± 10 ^abc^
Rub	9.51 ± 0 ^c^	2.23 ± 0 ^fg^	41.01 ± 4 ^b^	110.2 ± 9 ^c^	30.17 ± 2 ^fg^	451.5 ± 36 ^b^	990.1 ± 3 ^b^	453.9 ± 4 ^d^	584.2 ± 35 ^a^	375.2 ± 5 ^d^	335.2 ± 16 ^e^	150.8 ± 3 ^a^
Al K	6.46 ± 0 ^de^	4.6 ± 0 ^d^	20.73 ± 1 ^i^	80.35 ± 8 i	58.85 ± 5 ^d^	329.1 ± 23 ^efg^	847.9 ± 5 ^d^	351.3 ± 5 ^g^	371.5 ± 19 ^c^	266.4 ± 18 ^gh^	413.4 ± 23 ^bc^	66.52 ± 1 ^gh^
Mor	6.54 ± 0 ^de^	0.53 ± 0 ^i^	29.32 ± 2 ^e^	87.54 ± 5 ^g^	18.06 ± 1 ^i^	330.9 ± 16 ^efg^	770.1 ± 5 ^f^	123.2 ± 1 ^j^	179.9 ± 11 ^g^	299.3 ± 18 ^f^	23.81 ± 1 ^hi^	106.0 ± 2 ^bcdef^
Kras	2.53 ± 0 ^h^	1.33 ± 0 ^h^	27.75 ± 2 ^f^	39.03 ± 3 ^l^	14.06 ± 1 ^j^	268.6 ± 12 ^hi^	801.4 ± 5 ^e^	133.9 ± 2 ^j^	228.1 ± 14 ^f^	243.8 ± 9 ^h^	393.7 ± 27 ^cd^	99.64 ± 6 ^cdefg^
Kub	2.57 ± 0 ^h^	1.03 ± 0 ^hi^	24.81 ± 1 ^g^	43.71 ± 3 ^j^	30.52 ± 1 ^fg^	286.9 ± 24 ^h^	699.8 ± 5 ^h^	283.1 ± 4 ^h^	329.1 ± 29 ^d^	256.4 ± 15 ^h^	388.2 ± 34 ^cd^	80.03 ± 7 ^efg^
Oranz	2.84 ± 0 ^gh^	1.16 ± 0 ^h^	19.76 ± 1 ^j^	40.67 ± 3 ^k^	33.08 ± 2 ^fg^	172.1 ± 12 ^j^	666.0 ± 1 ^i^	247.4 ± 4 ^i^	180.4 ± 5 ^g^	239.1 ± 9 ^h^	53.10 ± 3 ^h^	43.87 ± 1 ^h^
Sahh	5.77 ± 0 ^e^	3.58 ± 0 ^e^	25.37 ± 2 ^g^	15.10 ± 1 ^p^	33.32 ± 2 ^f^	263.2 ± 15 ^hi^	756.1 ± 5 ^fg^	260.2 ± 5 ^hi^	396.0 ± 8 ^c^	293.85 ± 1 ^fg^	209.2 ± 16 ^f^	110.7 ± 10 ^bcde^
Vef	7.33 ± 0 ^d^	1.24 ± 0 ^h^	15.97 ± 1 ^l^	25.17 ± 1 ^n^	20.18 ± 1 ^hi^	317.6 ± 24 ^fg^	913.7 ± 7 ^c^	416.0 ± 7 ^ef^	209.9 ± 16 ^fg^	313.0 ± 10 ^e^	19.70 ± 1 ^i^	75.43 ± 7 ^fgh^
Ross	4.45 ± 0 ^f^	2.1 ± 0 ^g^	18.61 ± 1 ^k^	109.5 ± 5 ^d^	6.15 ± 0 ^k^	244.7 ± 18 ^i^	813.0 ± 4 ^e^	395.3 ± 2 ^ef^	293.1 ± 24 ^e^	380.4 ± 16 ^cd^	122.1 ± 2 ^g^	79.39 ± 2 ^bcdef^
Soln	8.64 ± 0 ^c^	3.8 ± 0 ^e^	28.3 ± 2 ^f^	91.73 ± 6 ^f^	32.36 ± 1 ^g^	324.5 ± 23 ^efg^	911.5 ± 7 ^c^	420.9 ± 4 ^ef^	321.9 ± 23 ^de^	406.5 ± 8 ^bc^	443.7 ± 39 ^a^	146.6 ± 9 ^a^
Ang	3.77 ± 0 ^g^	2.65 ± 0 ^f^	23.02 ± 2 ^h^	21.89 ± 2 ^o^	31.64 ± 1 ^fg^	286.9 ± 23 ^h^	728.9 ± 3 ^gh^	470.2 ± 3 ^cd^	297.2 ± 5 ^e^	329.4 ± 25 ^e^	215.5 ± 4 ^f^	117.5 ± 11 ^abcd^
Buss	2.81 ± 0 ^gh^	3.5 ± 0 ^e^	16.04± 1 ^l^	108.2 ± 5 ^e^	37.56 ± 1 ^e^	297.8 ± 22 ^g^	756.5 ± 4 ^fg^	420.2 ± 3 ^e^	369.6 ± 25 ^c^	259.4 ± 10 ^h^	119.6 ± 8 ^g^	84.62 ± 7 ^defg^
Rosi	5.29 ± 0 ^f^	1.1 ± 0 ^h^	21.12 ± 1 ^i^	31.60 ± 2 ^m^	6.03 ± 0 ^k^	332.5 ± 15 ^ef^	803.6 ± 8 ^e^	389.9 ± 2 ^f^	300.2 ± 14 ^de^	329.3 ± 28 ^e^	203.2 ± 12 ^f^	116.4 ± 8 ^abcd^
Wild	NA	1.49 ± 0 ^h^	31.7 ± 2 ^d^	NA	21.77 ± 1 ^h^	358.6 ± 24 ^e^	NA	470.7 ± 5 ^c^	313.2 ± 19 ^de^	NA	226.9 ± 16 ^f^	135.2 ± 4 ^ab^

Results are mean values of four replicate analyses calculated in mg GAE/g dw for TPC and µM TE/g dw for antioxidant capacity. NA—data not available; different letters on columns mark significant differences at *p* ≤ 0.05.

**Table 3:** The distribution of individual phenolic compounds (µg/g dw) in fruit, juice, and pomace extracts of 17 sweet rowanberry cultivars.

		Bur	Lik	Gran	Rub	Al_K	Mor	Kras	Kub	Oranz	Sahh	Vef	Ross	Soln	Ang	Buss	Rosi	Wild
NCha	F	3086 ± 781	4955 ± 323	2553 ± 182	2441 ± 231	1991 ± 132	1677 ± 131	1014 ± 164	991 ± 0.82	0.944 ± 0.073	1891 ± 141	1779 ± 162	1181 ± 103	2541 ± 182	1241 ± 122	1850 ± 183	1518 ± 142	1531 ± 101
	J	3930 ± 123	6127 ± 108	3497 ± 42	3402 ± 133	2475 ± 46	1830 ± 28	1122 ± 19	1176 ± 42	1023 ± 46	2122 ± 55	1963 ± 122	1289 ± 14	2040 ± 692	1461 ± 29	2297 ± 27	1942 ± 28	1813 ± 41
	p	1681 ± 61	2172 ± 182	1621 ± 50	1453 ± 92	1181 ± 43	1171 ± 21	862 ± 35	752 ± 21	713 ± 12	1281 ± 43	1472 ± 32	901 ± 12	2021 ± 44	981 ± 22	1641 ± 21	1204 ± 42	1392 ± 11
ChA	F	2013 ± 502	2269 ± 83	2011 ± 2.1	2440 ± 72	1052 ± 43	2662 ± 61	2636 ± 12	2700 ± 121	2448 ± 41	3789 ± 83	1982 ± 41	2692 ± 52	2028 ± 32	3031 ± 63	3142 ± 102	2450 ± 52	3312 ± 44
	J	2213 ± 59	2640 ± 59	2731 ± 102	3368 ± 140	1265 ± 60	2834 ± 216	3139 ± 145	3186 ± 126	2625 ± 122	4595 ± 106	2202 ± 147	3064 ± 126	2515 ± 129	3459 ± 76	3843 ± 209	3359 ± 95	3591 ± 154
	p	1564 ± 28	1620 ± 102	1994 ± 64	2261 ± 135	1070 ± 18	2745 ± 115	3232 ± 27	2782 ± 216	2669 ± 69	3622 ± 59	2205 ± 31	2894 ± 91	2477 ± 81	3161 ± 123	3639 ± 93	3024 ± 23	4782 ± 181
Cygal	F	5526 ± 602	4775 ± 263	2661 ± 122	2077 ± 73	716 ± 12.	274 ± 14	137 ± 20	158 ± 12	81 ± 1	118 ± 12	290 ± 13	132 ± 21	109 ± 12	147 ± 1	506 ± 22	279 ± 12	183 ± 1
	J	2627 ± 184	2704 ± 413	1884 ± 63	1288 ± 92	497 ± 13	235 ± 10	115 ± 4	146 ± 7	51 ± 12	94 ± 7	227 ± 20	126 ± 5	113 ± 8	134 ± 7	460 ± 13	273 ± 8	72 ± 12
	p	28 ± 2	26 ± 2	17 ± 2	9 ± 0	5 ± 0	2 ± 0	1 ± 0	1 ± 0	0	1 ± 0	2 ± 0	1 ± 0	1 ± 0	1 ± 0	3 ± 0	1 ± 0.0	2 ± 0
Cyglu	F	217 ± 42	175 ± 32	119 ± 11	141 ± 13	10 ± 2	18 ± 2	0	1 ± 0	0	0	10	1 ± 0	1 ± 0	1 ± 0	121 ± 2	25 ± 2	1 ± 0
	J	126 ± 4	127 ± 2	78 ± 1	110 ± 2	6 ± 0	18 ± 0	1 ± 0	1 ± 0	0	1 ± 0	1 ± 0	32 ± 2	1 ± 0	0	113 ± 2	23 ± 2	1 ± 0
	p	600 ± 31	554 ± 21	323 ± 53	362 ± 32	18 ± 2	27 ± 1	2 ± 0	66 ± 2	1 ± 2	1 ± 0	5 ± 0	2 ± 0	2 ± 0	2 ± 0	6 ± 0	36 ± 2	14 ± 2
Cyara	F	1538 ± 21	1380 ± 74.	424 ± 2.	60 ± 2	13 ± 2	5 ± 0	6 ± 0	6 ± 0	2 ± 0	4 ± 0	14 ± 1	5 ± 0	3 ± 0.0	6 ± 1	11 ± 2	5 ± 0	6 ± 0
	J	668 ± 57	690 ± 130	288 ± 8	37 ± 2	8 ± 0	3 ± 0	4 ± 0	5 ± 0	1 ± 0	3 ± 0	11 ± 2	4 ± 0	3 ± 0	5 ± 0	9 ± 0	4 ± 0	2 ± 0
	p	9 ± 0	8 ± 0	4 ± 0	1 ± 0	0	0	0	0	0	0	0	0	0	0	0	0	0
Ecat	F	27 ± 1	31 ± 2	27 ± 2	26 ± 2	22 ± 2	17 ± 1	28 ± 3	35 ± 2	27 ± 6	20 ± 2	13 ± 2	44 ± 2	13 ± 2	38 ± 6	13 ± 0	15 ± 2	33 ± 2
	J	10 ± 1	14 ± 1	16 ± 1	12 ± 1	11 ± 1	8 ± 1	16 ± 1	24 ± 1	14 ± 1	13 ± 0.1	8 ± 0.1	23 ± 0.1	8 ± 0.1	20 ± 0.1	07 ± 0	10 ± 0	12 ± 0
	p	59 ± 3	62 ± 3	69 ± 1	65 ± 3	65 ± 3	59 ± 2	70 ± 7	102 ± 3	84 ± 5	96 ± 3	65 ± 5	103 ± 1	54 ± 6	100 ± 2	53 ± 2	60 ± 3	111 ± 3
Cat	F	9 ± 2	9 ± 2	11 ± 2	26 ± 2	17 ± 2	19 ± 2	18 ± 2	22 ± 02	30 ± 2	19 ± 2	5 ± 2	23 ± 2	22 ± 2	25 ± 02	92	16 ± 2	17 ± 2
	J	5 ± 0	6 ± 0	8 ± 1	13 ± 2	8 ± 2	10 ± 1	12 ± 1	014 ± 1	016 ± 1	14 ± 1	3 ± 1	12 ± 1	14 ± 1	12 ± 1	6 ± 1	12 ± 1	8 ± 1
	p	21 ± 2	22 ± 2	37 ± 2	65 ± 2	48 ± 2	69 ± 2	44 ± 2	54 ± 2	82 ± 2	90 ± 2	22 ± 2	59±	93 ± 2	56 ± 0	29 ± 2	66 ± 2	52 ± 0
Coum	p	12 ± 1	9 ± 1	8 ± 1	24 ± 1	4 ± 0	5 ± 0	7 ± 1	9 ± 1	7 ± 1	6 ± 1	15 ± 1	15 ± 1	9 ± 1	17 ± 1	12 ± 1	8 ± 1	21 ± 1
Fer	p	10 ± 0	11 ± 0	8 ± 0	28 ± 0	4 ± 0	5 ± 0	7 ± 0	9 ± 0	5 ± 0	6 ± 0	23 ± 0	16 ± 0	7 ± 0	20 ± 0	25 ± 0	8 ± 0	10 ± 0
Q	F	50 ± 1	9 ± 1	9 ± 1	9 ± 1	5 ± 0	8 ± 1	6 ± 1	7 ± 1	5 ± 0	4 ± 0	8 ± 1	6 ± 1	9 ± 1	10 ± 1	7 ± 1	10 ± 1	12 ± 1
	J	14 ± 1	29 ± 1	28 ± 1	30 ± 1	23 ± 1	22 ± 1	23 ± 1	23 ± 1	18 ± 1	33 ± 1	22 ± 1	36 ± 1	37 ± 1	40 ± 1	25 ± 1	26 ± 1	17 ± 1
	p	58 ± 1	41 ± 1	73 ± 1	82 ± 1	37 ± 1	45 ± 1	35 ± 1	29 ± 1	30 ± 1	55 ± 1	131 ± 1	69 ± 1	64 ± 1	84 ± 1	63 ± 1	75 ± 1	121 ± 1
Qgal	F	268 ± 34	199 ± 7	220 ± 8	89 ± 11	119 ± 12	94 ± 6	48 ± 1	63 ± 1	40 ± 4	426 ± 36	185 ± 7	68 ± 1	238 ± 10	108 ± 4	53 ± 5	91 ± 1	232 ± 1
	J	199 ± 6	163 ± 18	202 ± 8	87 ± 5	89 ± 4	51 ± 1	45 ± 1	31 ± 1	23 ± 1	357 ± 26	129 ± 15	46 ± 1	207 ± 9	75 ± 1	47 ± 1	85 ± 1	63 ± 1
	p	338 ± 6	627 ± 18	270 ± 8	138 ± 5	187 ± 9	133 ± 1	111 ± 3	69 ± 1	64 ± 3	619 ± 4	345 ± 2	708 ± 1	361 ± 8	129 ± 1	72 ± 4	155 ± 4	741 ± 1
Qglu	F	289 ± 13	222±	200 ± 7	115 ± 9	188 ± 23	78 ± 3	28 ± 1	35 ± 3	34 ± 3	27,838	62 ± 3	37 ± 1	617 ± 17	53 ± 3	20 ± 1	74 ± 1	143 ± 3
	J	226 ± 17	191 ± 20	200 ± 5	125 ± 0	146 ± 1	47 ± 0	25 ± 1	18 ± 2	20 ± 9	231 ± 1	46 ± 1	27 ± 4	132 ± 1	34 ± 3	17 ± 4	74 ± 2	43 ± 1
	p	344 ± 15	277 ± 37	240 ± 21	183 ± 44	300 ± 13	115 ± 7	61 ± 4	41 ± 1	55 ± 9	403 ± 18	12,418	42 ± 0	241 ± 5	63 ± 6	33 ± 1	125 ± 25	431 ± 71
Qrut	F	246 ± 27	240 ± 14	166 ± 5	224 ± 15	242 ± 15	91 ± 1	82 ± 1	122 ± 7	95 ± 7	0	195 ± 13	115 ± 1	242 ± 6	165 ± 5	23 ± 0	84 ± 1	73 ± 1
	J	232 ± 6	245 ± 13	164 ± 3	229 ± 10	205 ± 5	60 ± 2	57 ± 2	47 ± 2	50 ± 2	0	135 ± 6	64 ± 1	214 ± 2	84 ± 2	19 ± 1	83 ± 2	18 ± 2
	p	819 ± 10	225 ± 8	118 ± 3	202 ± 7	255 ± 20	70 ± 1	124 ± 5	73 ± 4	84 ± 4	1 ± 0	221 ± 6	61 ± 3	25,715	100 ± 5	21 ± 1	74 ± 1	221 ± 1
Ka	F	7 ± 1	5 ± 0	5 ± 0	6 ± 1	6 ± 1	6 ± 1	2 ± 0	4 ± 0	30	15 ± 1	2 ± 0	3 ± 0	8 ± 1	5 ± 0	1 ± 0	61	9 ± 1
	J	50	60	6 ± 0	70	5 ± 0	3 ± 0	2 ± 0	2 ± 0	2 ± 0	12 ± 0	1 ± 0	2 ± 0	6 ± 0	3 ± 0	1 ± 0	4 ± 0	2 ± 0
	p	3 ± 0	3 ± 0	4 ± 0	7 ± 0	2 ± 0	5 ± 0	4 ± 0	4 ± 0	3 ± 0	4 ± 0	7 ± 0	9 ± 0	4 ± 0	10 ± 0	4 ± 0	7 ± 0	10 ± 0
Isor	p	7 ± 0	7 ± 0	5 ± 0	21 ± 0	5 ± 0	1 ± 0	1 ± 0	0	0	0	2 ± 0	1 ± 0	1 ± 0	1 ± 0	1 ± 0	1 ± 0	1 ± 0
PCA	p	46 ± 6	27 ± 4	40 ± 3	128 ± 10	8 ± 0	4 ± 0	4 ± 0	5 ± 0	5 ± 0	4 ± 0	17 ± 0	11 ± 0	4 ± 0	15 ± 0	18 ± 2	13 ± 1	17 ± 0
Caf	p	24 ± 1	18 ± 2	24 ± 3	67 ± 7	13 ± 1	16 ± 1	20 ± 1	23 ± 3	21 ± 1	21 ± 2	34 ± 1	51 ± 2	17 ± 1	51 ± 2	44 ± 2	34 ± 2	38 ± 2
P_B1	F	8 ± 1	10 ± 1	16 ± 1	40 ± 4	22 ± 1	26 ± 1	24 ± 5	28 ± 5	40 ± 2	26 ± 5	6 ± 1	31 ± 1	30 ± 1	32 ± 1	12 ± 1	21 ± 1	22 ± 1
	J	3 ± 0	5 ± 0	8 ± 1	18 ± 1	10 ± 1	14 ± 1	16 ± 1	18 ± 1	21 ± 1	19 ± 1	4 ± 0	18±	22 ± 1	17 ± 1	6 ± 0	14 ± 1	10 ± 1
	p	24 ± 1	25 ± 2	42 ± 4	75 ± 3	51 ± 2	72 ± 3	49 ± 2	60 ± 1	98 ± 9	99 ± 7	24 ± 3	68 ± 7	100 ± 10	61 ± 4	32 ± 3	68 ± 5	57 ± 1
P_B2	F	79 ± 1	94 ± 0	4 ± 7	80 ± 8	66 ± 8	49 ± 4	75 ± 1	97 ± 1	78 ± 1	60 ± 1	41 ± 1	118 ± 1	36 ± 1	107 ± 2	42 ± 5	44 ± 1	92 ± 1
	J	28 ± 5	40 ± 7	49±	42 ± 1	33 ± 5	23 ± 6	47 ± 1	71 ± 2	36 ± 1	42 ± 3	24 ± 1	66 ± 2	28 ± 1	62 ± 3	25 ± 4	29 ± 3	33±
	p	162 ± 7	172 ± 7	186 ± 7	185 ± 10	176 ± 16	150 ± 14	188 ± 13	259 ± 6	209 ± 12	255 ± 18	174 ± 10	293 ± 7	139 ± 22	271 ± 19	145 ± 4	164 ± 3	306 ± 1
P_C1	F	93 ± 1	105 ± 1	88 ± 1	96 ± 1	78 ± 1	53 ± 1	77 ± 1	100 ± 1	83 ± 1	70 ± 1	40 ± 1	142 ± 1	49 ± 1	124 ± 1	39 ± 2	55 ± 1	103 ± 2
	J	34 ± 1	44 ± 10	53 ± 11	38 ± 11	36 ± 12	26 ± 0	41 ± 1	66 ± 1	43 ± 1	41 ± 1	26 ± 10	69 ± 12	26 ± 13	67 ± 11	20 ± 11	40 ± 12	41 ± 11
	p	187 ± 24	201 ± 7	222 ± 24	234 ± 11	202 ± 18	163 ± 3	187 ± 14	267 ± 32	240 ± 25	273 ± 5	189 ± 9	308 ± 44	188 ± 0	282 ± 24	150 ± 5	185 ± 4	312 ± 23

NCha—neochlorogenic acid; ChA—chlorogenic acid; Cy_gal—cyanidin-3-galactoside; Cy.glu—cyanidin-3-O-glucoside; Cy_ara—cyanidin-3-arabinoside; Ecat—epicatechin, Cat—catechin, Coum—p-coumaric acid, Caf—caffeic acid, Fer—ferulic acid, Ka—kaempferol, Isor—isorhamnetin, Q—quercetin, Qgal—quercetin-3-O-galactoside; Qglu—quercetin 3-glucoside; Qrut—quercetin-3-O-rutinoside; and P_B1, P_B2, and P_C1—procyanidins B1, B2, and C1.

**Table 4:** Correlation coefficients (*R*^2^) between the content of different groups of polyphenolic compounds and the antioxidant capacity of 16 *Sorbus* fruit, juice, and pomace extracts.

	TPC			HCA			ACY			FLAVO		
Part	F	J	P	F	J	P	F	J	P	F	J	P
ABTS	0.872	0.723	0.749	0.537	0.558	0.105	0.751	0.751	0.820	0.658	0.591	0.491
DPPH	0.547	0.948	0.810	0.221	0.616	0.188	0.527	0.893	0.886	0.278	0.658	0.514
ORAC	0.822	0.493	0.708	0.512	0.265	0.289	0.685	0.476	0.517	0.652	0.567	0.466

TPC—total phenolic content, HCA—hydroxycinnamic acids, ACY—anthocyanins, FLAVO—flavanols, F—fruit, J—juice, P—pomace.

## Data Availability

The data presented in this study are available in article.
